# Notes and News

**Published:** 1904-08-20

**Authors:** 


### Notes and News.

What is described as a relatively benign form of cholera has made its appearance in St. Petersburg, where it has caused much alarm. Severe measures are being adopted to stay the epidemic.

In connection with the sanitary regulations for the army a scheme is being revised whereby more responsibility in the supervision of sanitary services in barracks and camps will be placed upon non-medical officers.

We regret to record the death of Sir Frederic Bateman, M.D. He was chiefly distinguished for his writings on mental subjects, and was a member of numerous foreign medical and learned societies. He was knighted in 1892.

A large British Hospital, to be administered by English Blue Nuns, is being established on the Cœlean Hill. The foundation-stone will be laid in October by Cardinal Respighi. It will be under English patronage and protection.

The late Mr. Thomas Whiffen, of Wandsworth, has bequeathed £500 each to St. George's, St. Thomas's, Guy's, Charing Cross, the Middlesex Hospital, and the Victoria Hospital for Children, and £250 each to numerous other hospitals and charities.

A combination hospital for the Eastern District of Haddingtonshire and the Burgh of Dunbar has been opened at Belshaven. It consists of two pavilions, each containing two wards, and of an administration block, mortuary, and other adjuncts. The cost was £8,732.

The St. Mary's and the Southern Hospitals at Manchester have amalgamated, and the institutions will be called the St. Mary's Hospitals. This amalgamation will provide an excellent midwifery training school for students and midwives. The Southern Hospital will provide a new hospital, with a department for the diseases of women and children. Some years ago a magnificent gift was offered the two hospitals if they would amalgamate, but was lost by St. Mary's Hospital refusing to consent.

Miss Margaret Keith and Miss Isabella Aytoun have bequeathed £1,378 to the Edinburgh Royal Infirmary.

Dr. Lorrain Smith, Musgrave Professor of Pathology at Queen's College, Belfast, has been appointed Professor of Pathology at the Victoria University, Manchester. He received his medical education at Edinburgh.

Mr. George Herring has sent a donation of £509 to the North-West London Hospital, Kentish Town Road, being the promised one-third of the total amount (£1,527) subscribed in response to Lord Rathmore's recent appeal.

The Portsmouth people have approved of the Royal Portsmouth Hospital Enlargement Fund, and it is to be hoped that they will now see to it that the Fund is a success. The present 120 beds are found inadequate for a district with 240,000 people. In the proposed new wing there will be 42 beds, and the cost will be £10,000. An operating-theatre is another urgent need. Out of the £10,000 over £3,000 is promised or in hand.

By way of a testimonial to Mr. Alfred Willett, on his retirement from the post of surgeon at St. Bartholomew's Hospital, £250 has been subscribed. This sum is to be devoted to the foundation of a "Willett Medal," to be awarded each year to the candidate for the Brackenbury Surgical Scholarship who obtains the highest marks in operative surgery. The medal, which is in silver, bears a portrait of Mr. Willett on one side, and a representation of the hospital and of the hospital arms on the other.

At the last meeting of the Portsmouth Workhouse Infirmary a report dealing with the question of providing more accommodation for operations caused considerable discussion. The medical officer maintained that up to the present time the accommodation had been ample. Mrs. Hawksley put forward definite complaints as to the manner in which the reports on operations were kept and the general system of conducting operations, and she advocated the building of an operating-theatre. However, the majority of the meeting was in favour of the present arrangements.

A certain amount of indignation has been expressed in the Press against the action of the Hospital Sunday Fund, because it has withheld a grant from the City Orthopædic Hospital. It seems a pity that the public should be led by sentiment to hastily condemn the action of a body of men so experienced and well informed in hospital matters as the managers of the Metropolitan Hospital Sunday Fund. The majority of the public have neither experience nor opportunity to test the individual merits of the institutions. The power to judge of the proper standard of economy, efficiency, and comfort for the patients is not learnt in a day, and those who would give wisely are fortunate that they can obtain the assistance of the collective experience of the Hospital Sunday, Saturday, and the King's Fund to secure that the maximum benefit to the sick poor shall result from their gifts. It is as easy to misspend money in the direction of charity as in any other.

